# Toward Improving Electrocardiogram (ECG) Biometric Verification using Mobile Sensors: A Two-Stage Classifier Approach

**DOI:** 10.3390/s17020410

**Published:** 2017-02-20

**Authors:** Robin Tan, Marek Perkowski

**Affiliations:** Department of Electrical and Computer Engineering, Portland State University, Portland, OR 97201, USA; rtan@pdx.edu

**Keywords:** electrocardiogram (ECG), biometric recognition, random forest, wavelet distance measure, data security

## Abstract

Electrocardiogram (ECG) signals sensed from mobile devices pertain the potential for biometric identity recognition applicable in remote access control systems where enhanced data security is demanding. In this study, we propose a new algorithm that consists of a two-stage classifier combining random forest and wavelet distance measure through a probabilistic threshold schema, to improve the effectiveness and robustness of a biometric recognition system using ECG data acquired from a biosensor integrated into mobile devices. The proposed algorithm is evaluated using a mixed dataset from 184 subjects under different health conditions. The proposed two-stage classifier achieves a total of 99.52% subject verification accuracy, better than the 98.33% accuracy from random forest alone and 96.31% accuracy from wavelet distance measure algorithm alone. These results demonstrate the superiority of the proposed algorithm for biometric identification, hence supporting its practicality in areas such as cloud data security, cyber-security or remote healthcare systems.

## 1. Introduction

Mobile devices are now indispensable in our daily lives in social networking, ecommerce, online banking, and remote healthcare. As a result, a large amount of personal data is stored in the cloud and can be accessed anywhere around the globe. This poses a major concern in data security and confidentiality [1,2]. Traditional biometric recognition systems based on fingerprints or iris exhibit certain limitations regarding robustness against falsified credentials or spoof attacks [3,4]. To overcome the existing challenges, electrocardiograms (ECGs) have been studied as an emerging biometric modality for subject verification because of their high distinctiveness, difficult replication, and intrinsic aliveness detection [5,6]. Conventionally, ECG tests rely on a set of 12 leads placed on a human body in a clinical environment. More recently, low-power and small-size biosensors are being integrated into mobile devices for real-time monitoring of people’s physiological conditions during daily activities, including electrocardiograms. This makes mobile ECG data readily available for biometric recognition without added hardware cost.

**Figure 1:**
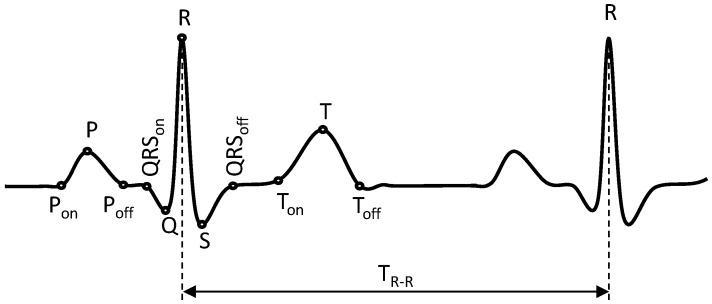
Electrocardiogram (ECG) P-QRS-T complex and fiducial characteristic points.

The use of ECG for identity recognition dates back to the pioneer studies of Biel et al. [7], Irvine et al. [8], and Kyoso and Uchiyama [9], which revealed that ECG contains sufficiently detailed information to uniquely identify an individual. During the last decade, considerable efforts have been made to develop various algorithms using ECG for subject identification [10,11,12,13,14,15]. Generally speaking, the ECG identification systems are categorized based on the method of feature (also called template) extraction as well as the type of template matching for classification. The ECG feature extraction methods are mainly categorized as either fiducial-based or non-fiducial-based. The fiducial-based feature extraction relies on an accurate detection of ECG fiducial characteristic points such as P, Q, R, S, T, P_on_, P_off_, QRS_on_, QRS_off_, T_on_, and T_off_ as shown in Figure 1, to obtain their relative amplitude, temporal intervals and morphological features [5,6,7,9,16,17,18]. Here P_on_, P_off_, QRS_on_, QRS_off_, T_on_, and T_off_ represent the onset and offset timing location of P-wave, QRS-wave, and T-wave (Figure 1). The non-fiducial-based feature extraction analyzes the ECG complexes (of heartbeats) using time or frequency analysis such as discrete wavelet transform (DWT) or discrete cosine transform (DCT) to obtain other statistical features [10,14,19,20]. The template-matching classifiers include one-to-many template matching [5,9,10], *k*-nearest neighbor algorithms (KNN) [21,22], and non-linear machine learning techniques such as the artificial neural networks (ANN) [23] and support vector machines (SVM) [24]. 

The ECG subject verification performance reported by the literature varies depending on databases, methods of ECG data recording, sample size, signal pre-processing techniques, types of feature extraction, and classification methods. Many studies were based on the clinical multi- or single-lead ECG data, such as MIT-Beth Israel Hospital (MIT-BIH) and Physikalisch-Technische Bundesanstalt (PTB) diagnostic databases hosted on the Physionet website [25,26,27,28,29,30,31,32]. A few other studies built the sensor design into a lab prototype to obtain “off-the-person” ECG data for analyzing the performance of ECG biometric identification. For instance, Shen et al. [6] implemented the identity verification using template matching and decision-based neural network (DBNN) for a group of 20 subjects from the MIT-BIH database, and reported the rate of correct identity verification as 95% for template matching and 80% for the DBNN, while combining the two methods produced a 100% correct rate. Wang et al. [33] developed a hierarchical architecture integrating human ECG fiducial features and appearance features for classification, and achieved 100% correct human identification when evaluated with 13 subjects. Shen et al. [11] applied a combined template matching and distance classification methods to the ECG signal recorded from the palms for biometric recognition for a group of 168 subjects, and achieved a 95.3% identification rate. Chan et al. [10] studied the biometric performance of one-channel ECG signals recorded from the pads of individuals’ thumbs, from a group of 50 subjects during three data-recording sessions on different days, and achieved 89% classification accuracy using a wavelet distance measure classifier. For more details, we suggest the review paper of ECG biometric recognition by Odinaka et al. [34].

The latest advancement of integrating biosensors into mobile devices has facilitated the real-time measurement of ECG signal for monitoring human health conditions [3]. Taking advantage of the fact that mobile ECG data might be readily available, we aimed to develop a robust and effective mobile ECG biometric recognition system for reliable access control in different applications. Compared to the ECG data measured from a multi-lead clinical setup, the mobile ECG signals suffer from poorer signal-to-noise ratio and baseline drift due to human respiration or motion artifacts from finger to electrode pad contact, as well as power line interferences [35,36,37]. As a result, the detections of mobile ECG wave boundaries (i.e., onset and offset points) become less reliable as they are mostly susceptible to noise and baseline wandering, leading to a sub-optimal performance using fiducial-based feature extraction methods. Alternatively, although non-fiducial-based feature extraction methods obviate the need for detecting ECG fiducial points, the subject verification accuracy varies depending on the ECG signal quality as well as the choice of classification method. Moreover, when the subject size magnifies, the performance tends to degrade and computational load increases significantly [34,38,39].

To overcome the mobile ECG limitations and improve the accuracy and robustness of a biometric recognition system using mobile ECG, a new two-stage subject verification system is proposed in this paper that takes the advantages of both fiducial and non-fiducial features and intelligently combines a probabilistic random forest classifier with a one-to-many template matching classifier based on wavelet coefficients. To objectively assess the performance of the proposed algorithm, a new ECG database is created by combining ECG data from four sources, including the ECG from a mobile phone, the ECG in the presence of arrhythmia, the ECG with normal sinus rhythm, and the ECG data measured over a 6-month span. To the best of our knowledge, our method is the first study to use a prototype system of industrial sensors integrated into mobile phones to obtain ECG data. An up/down sampling technique is utilized to re-sample the ECG signals at different sampling rates into a uniform one, to ensure the proper operation of the two-stage classification system.

The rest of this paper is organized as follows. The proposed ECG biometric verification system is presented in Section 2; Section 3 describes the ECG data sources as well as the generation of the new database using cubic spline interpolation; the methodology of the proposed algorithm is presented in detail in Section 4; the results and discussions are demonstrated in Section 5; finally, Section 6 concludes the paper.

## 2. System Framework

**Figure 2:**
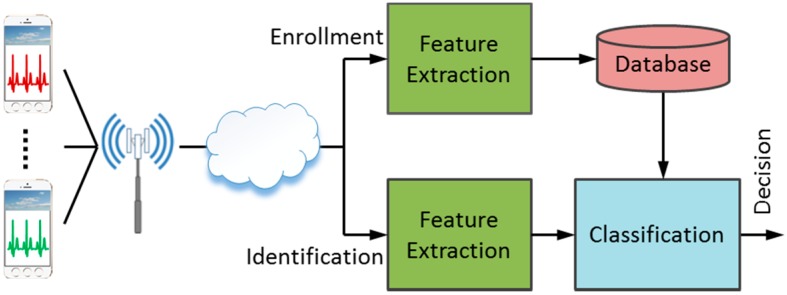
Biometric patient verification system using mobile ECG.

**Figure 3:**
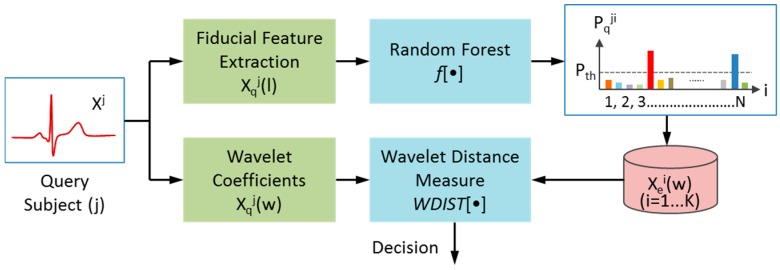
Proposed two-stage ECG biometric subject verification algorithm. The fiducial feature $X_{q}^{j}\left(l\right)$ is first applied to the random forest classifier, to identify the limited K potential candidates (usually K<5). Next, the non-fiducial feature $X_{q}^{j}\left(w\right)$ (wavelet coefficient) is applied to a 1-to-K template matching classifier to make the final decision.

Figure 2 shows the framework of a typical biometric verification system using mobile ECG. The system operates in two stages: the enrollment stage and the verification stage. The ECG signal taken from an individual by a mobile device is transmitted to the biometric verification system in a remote center over wireless networks. At the time of enrollment, the system extracts a set of features from the ECG signal of each individual, and stores the feature template into the database. During the verification stage, when the ECG signal from an unknown subject is received, the system again extracts a set of features using the same methodology and applies them to a classifier for decision making. In this study, a new two-stage verification algorithm is proposed, as illustrated in Figure 3.

From Figure 3, in the enrollment stage the ECG signal received from each individual i (i=1, … N) is first pre-processed, where N is the total number of individuals registered in the system. The system then extracts the fiducial features $X_{e}^{i}\left(l\right)$ based on limited ECG significant points (P, Q, R, S, T), as well as non-fiducial features $X_{e}^{i}\left(w\right)$ based on significant wavelet coefficients, and next stores both feature sets into the database. When the ECG signal from an unknown subject *j* is received, the system once again extracts its fiducial features $X_{q}^{j}\left(l\right)$ and non-fiducial features $X_{q}^{j}\left(w\right)$. Next, the proposed two-stage subject verification system includes the following steps: (1) application of the $X_{q}^{j}\left(l\right)$ feature set to a random forest classifier with function f[·]. The probabilities $P_{q}^{ji}$ for the unknown subject j being identified as the individual *i* in the database are then derived as given in Equation (1):

(1) $$P_{q}^{j1},\;P_{q}^{j2},\;\ldots ,\;P_{q}^{jN}=f\left[X_{q}^{j}\left(l\right)|X_{e}^{1}\left(l\right),\;X_{e}^{2}\left(l\right),\;\ldots ,\;X_{e}^{N}\left(l\right)\right]$$

(2) selection of K candidate subjects (K≪N) whose probabilities are higher than a pre-determined probability threshold $P_{th}$ and application of the data of those selected K subjects to the subsequent one-to-many template matching classifier; (3) use of a 1-to-K template matching classifier, based on significant wavelet coefficients, to calculate the wavelet distance D using the unknown identity feature $X_{q}^{j}\left(w\right)$ against each of the selected candidate subjects $X_{e}^{k}\left(w\right)$ (k=1, …, K) as given in Equation (2):

(2) $$D_{k}=WDIST\left[X_{q}^{j}\left(w\right)|X_{e}^{k}\left(w\right)\right],\;k=1,\ldots \;K$$

Here WDIST[·] is the function that calculates the distance between the test subject $X_{q}^{j}\left(w\right)$ and each candidate $X_{e}^{k}\left(w\right)$. Finally, a decision is made using the minimum $D_{k}$ which means that the candidate $X_{e}^{k}\left(w\right)$ with the smallest distance to $X_{q}^{j}\left(w\right)$ is selected. Compared to a conventional 1-to-N template matching classifier, the proposed two-stage classifier using 1-to-K template matching eliminates the concerns of significantly increased computational load and performance degradation as subject size N increases [34,38,39]. 

## 3. Data Sources

To support real-life practicality of biometric subject verification, the proposed algorithm is evaluated using multiple types of ECG data on subjects under different health conditions. Table 1 summarizes the database used, including a comparatively large database generated in this study.

**Figure 4:**
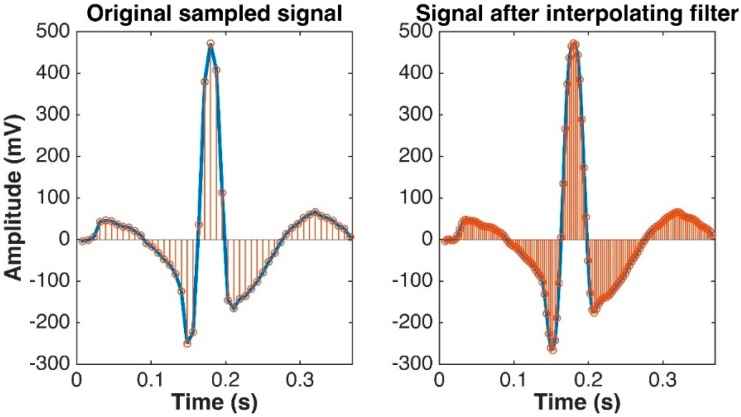
ECG up-sampling using cubic spline data interpolation. Left: 128 Hz; Right: 360 Hz.

A majority of previously developed ECG biometric recognition systems utilize ECG data measured from the same device at the same sampling rate. However, in real applications, the ECG signals may be taken from different types of mobile devices with different sampling frequencies. The unmatched sampling rate may lead to an improper operation of a classification system. To resolve this problem, an up/down-sampling interpolation technique is introduced in this study to convert the ECG signals at different sampling rates into a uniformed sampling rate (360 Hz is selected in this study). A unified database is then created by integrating all four datasets together using cubic spline data interpolation method for data up/down-sampling. Figure 4 illustrates an example of up-sampling using the cubic spline interpolation method for ECG data from the MIT-BIH normal sinus rhythm.

## 4. Methodology

Following Section 2 on the framework of the proposed ECG biometric subject verification system using combined random forest and wavelet distance measure classifiers, this section describes the methodology in detail for identifying an unknown subject.

### 4.1. Data Pre-Processing 

The raw ECG data is first applied to a bandpass filter using fast Fourier transform (FFT). The low frequency cutoff of the bandpass filter is set to 2 Hz to get rid of baseline wandering; the high frequency cutoff of the bandpass filter is set to 50 Hz to keep as much ECG signal energy as possible while removing the power line interference (60 Hz) and other high frequency noise.

### 4.2. R-Peak Detection

R-peak detection is the key to ensure each P-QRS-T complex is correctly delineated. The goal for R-peak detection is to locate the timing position for all true positive R-peaks while eliminating false positive R-peaks. In this study, a modified R-Peak detection is created. It combines the valley–peak detection algorithm with shifting windowing from [40] with an adaptive threshold algorithm as proposed by Pan and Tompkins [41]. The accuracy of this new R-peak detection algorithm is evaluated using the MIT-BIH arrhythmia database as the R-peak positions are accurately annotated by the medical staff. The proposed R-peak detection algorithm achieves a 99.46% true positive rate (TPR), which demonstrates the effectiveness of the modified R-peak detection algorithm. The computation time is 0.53 s, which is ~1/5 of the time needed by the Pan–Tompkins algorithm under the same development environment (the same computer device, with dual CPU cores of Intel^®^ core™-i5 processor 3.6 GHz).

### 4.3. P-QRS-T Complex Delineation 

**Figure 5:**
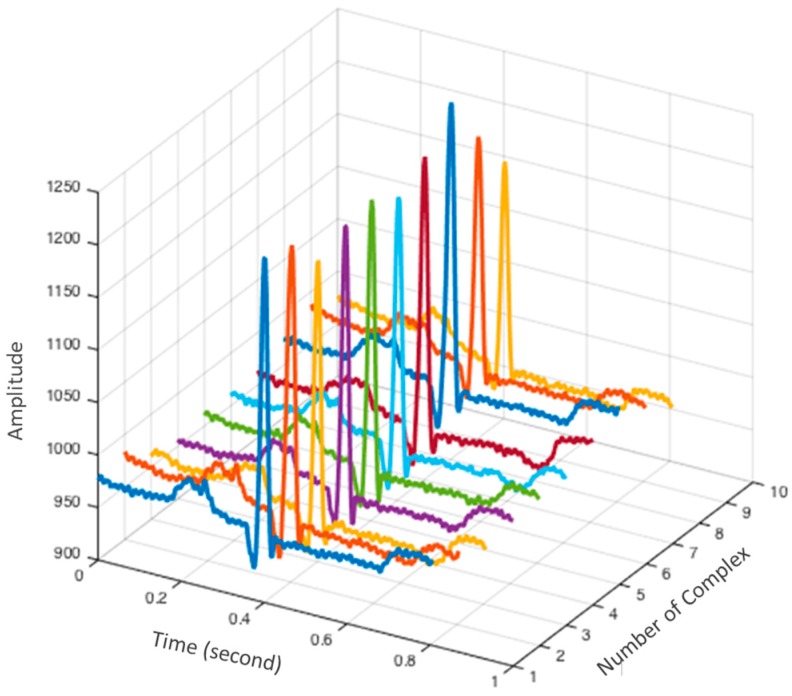
3D array of P-QRS-T complexes.

After R-peak detection, the next step is to delineate the P-QRS-T complex through time windowing for ECG feature extraction. In this study, a time window of 800 ms centered on R-peak location is used to segment each P-QRS-T complex. The 3D array of the delineated ECG complex is illustrated in Figure 5.

To further improve the feature extraction accuracy, some of the P-QRS-T outliers are removed as suggested by Chan et al. [10] by calculating the Pearson correlation coefficients for each P-QRS-T complex against the mean complex calculated from the 3D array. Figure 5 presents an example of 10 waveforms, and we define the mean complex as the mean of these waveforms. The distribution of the correlation coefficients is examined as follows. A threshold is determined as: μ−0.5×σ, where μ is the mean value of the correlation coefficient and σ is the standard deviation. If the correlation coefficient of a P-QRS-T complex falls below this pre-determined threshold, it is considered as an outlier and is removed.

### 4.4. Fiducial Feature Extraction

**Figure 6:**
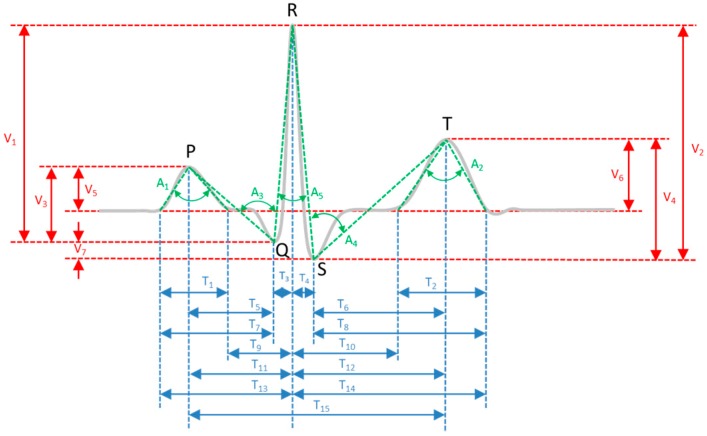
Feature extraction based on fiducial points.

Once the R point is detected, the P, Q, S, T peaks and valleys are first detected using a local maximum/minimum searching algorithm within a defined physical region. In this study, the Q and S points are limited within the 150 ms width window, centered at the R point. The P point is within a 200 ms period advance from the R point. The T point is within a 400 ms period backward from the R point. Next, the onset and offset points for P wave (P_on_, P_off_) and T wave (T_on_, T_off_) are determined using the triangle optimization method (for details please refer to Singh et al. [14]). Once those ECG significant points are correctly identified, the fiducial features are then extracted based on their relative temporal interval, amplitude, as well as angles of the ECG wave, as illustrated in Figure 6. Prior to data processing by the proposed patient verification system, the QT temporal intervals $T_{i}$ are scaled according to the Framingham formula [42]:

(3) $$T_{i}^{scaled}=T_{i}+0.154\times \left(1-T_{r-r}\right)$$

where $T_{r-r}$ is the time interval between the adjacent R peaks.

To achieve an optimized performance, three combinations of ECG significant points are investigated for feature extraction. Table 2 shows a summary of the extracted fiducial features from the three combinations of ECG fiducial points. Since the mobile ECG signals suffer from a poorer signal-to-noise ratio and baseline drift due to human respiration or motion artifacts from finger-to-electrode pad contact, the detections of mobile ECG wave boundaries (i.e., onset and offset points) become less reliable as they are mostly susceptible to noise and baseline wandering. Therefore, the presented two-stage cascaded method allows for fiducial features extracted from only P, Q, R, S, T peaks and valleys to be sufficient for subject verification. 

### 4.5. Non-Fiducial Feature Extraction

**Figure 7:**
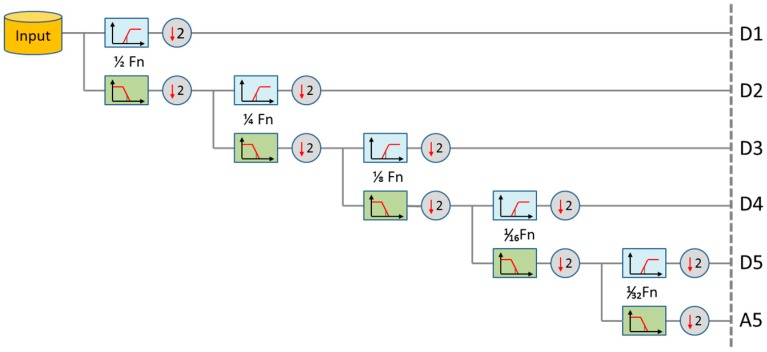
The 5-level decomposition using Daubechies discrete wavelet transform (DWT).

The ECG non-fiducial features are obtained based on DWT. The wavelet analysis provides a time–frequency representation of an analyzed signal x(t) in time domain, allowing a higher temporal resolution for high frequency components of x(t) and lower temporal resolution for its low-frequency components. This multi-scale time resolution is beneficial for ECG complex data processing, as the ECG waveform exhibits both high frequency data transitions (related to QRS) as well as low frequency waves (P, T) within a small P-QRS-T time window. Using the wavelet analysis algorithm, the original ECG data is hierarchically decomposed into N-level sub-series at different frequency bands. This is done by processing the input ECG data x(t) with two complementary high pass and low pass filters in a tree-structured fashion and down-sampling by two at each stage in order to decompose into a set of orthogonal components (D1 to D5, A5), as shown in Figure 7. In Figure 7, D1 to D5 represent the detailed time series and A5 represents the approximate time series at different frequency sub-bands; $F_{n}$ is the Nyquist frequency [43] of the ECG data. 

**Figure 8:**
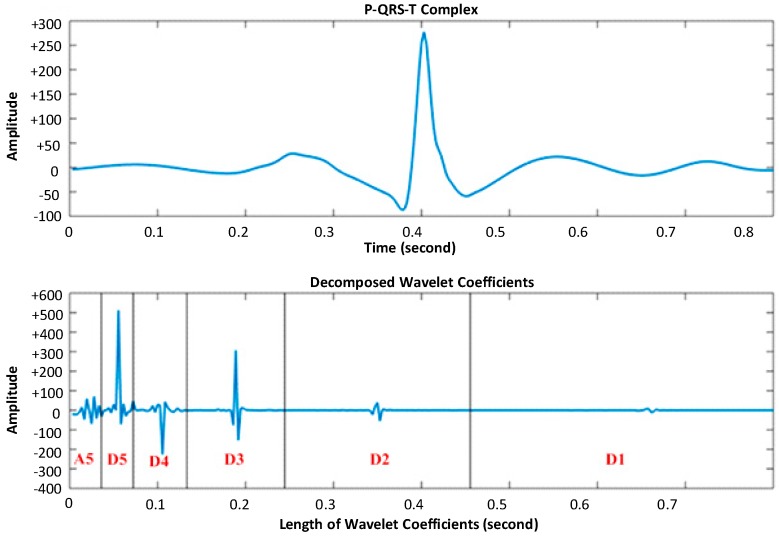
ECG original complex (top); and decomposed signal (bottom).

The corresponding wavelet coefficients $\omega_{pr}$ at each level of decomposition can be derived by Equation (4):

(4) $$\omega_{pr}=\int_{-\infty }^{+\infty }x\left(t\right)*\frac{1}{\sqrt{2^{p}}}\varphi \left(\frac{t-r*2^{p}}{2^{p}}\right)dt$$

where φ(t) is the selected mother wavelet, *p* is the scale parameter which represents the level of decomposition, and *r* is the shifting parameter which gives the number of wavelet coefficients at decomposition level *p*. In this study, the Daubechies (*db*) mother wavelet is used, which provides a family of wavelets called dbN, where N is the order of wavelets. For achieving the best overall patient verification accuracy, db3 is used to decompose each ECG P-QRS-T complex into sub-series *D*1 to *D*5 and *A*5, as illustrated in Figure 8.

For ECG data with sampling frequency $F_{s}$, its Nyquist frequency $F_{n}=F_{s}/2$. The relationship between the sampling frequency $F_{s}$ and the frequency bandwidth range $F_{p}$ at the p level wavelet decomposition is derived in Equation (5) as:

(5) $$\frac{F_{s}}{2^{p+1}}\leq F_{p}\leq \frac{F_{s}}{2^{p}}$$

Taking the MIT-BIH arrhythmia database as an example ($F_{s}=360\;\mathrm{Hz}$), Table 3 shows the frequency sub-band range and the number of wavelet coefficients at each level of wavelet decomposition. Considering the fact that ECG signal is pre-processed by a bandpass filter from 2 Hz to 50 Hz, the ECG non-fiducial features will only take the wavelet coefficients at the significant level of decomposition, within the frequency range of interest. 

### 4.6. Two-Stage Subject Verification System 

**Figure 9:**
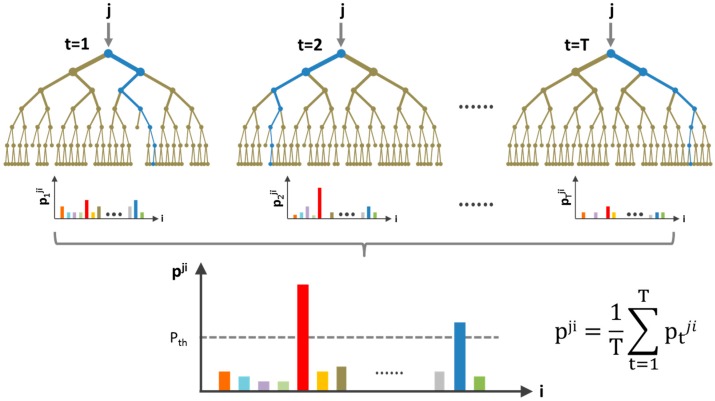
Random forest classifier for fiducial-based features. j represents an unknown subject; T is the total number of decision trees used in the random forest algorithm; N is the total number of subjects stored in the database; and $P^{ji}$ is a probability vector with size N, indicating j as being classified as any subject i (i=1,…N) in the database.

To enhance the robustness of the biometric subject verification system using mobile ECG, a two-stage automatic subject verification algorithm is developed. The ECG fiducial features are first applied to a random forest ensemble learning classifier. A random forest operates by constructing a multitude of independent decision tree predictors. A bootstrapping technique is used to resample the training datasets such that each decision tree takes only a subset of input data. During the model training process, each leaf node of the decision tree produces estimates of several conditional class probabilities. When an input subject arrives at this leaf node, it gives the probability of being classified as any subject i (i=1, … N) in the database. Figure 9 shows the decision-making process when an unknown subject *j* is applied to the random forest classifier.

**Figure 10:**
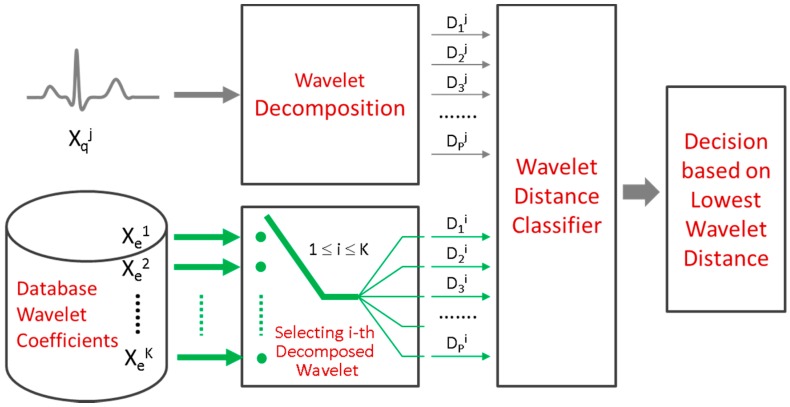
1-to-K template matching using wavelet distance measure.

In Figure 9, T is the total number of decision trees used in the random forest algorithm; N is the total number of subjects stored in the database. The final classification probability of the random forest classifier is the average of the probabilities from the terminal nodes of all decision trees that a test subject has reached. Only a few candidate subjects K whose probability is above an optimized probability threshold $P_{th}$ will be applied to the subsequent 1-to-K template matching classifier for further decision. The probability threshold $P_{th}$ is determined based on the overall subject verification accuracy. Unlike a conventional 1-to-N template matching classifier where the feature vector of an unknown subject is compared to that of all N elements of the subject database, the proposed algorithm only needs to implement 1-to-K template matching, as shown in Figure 10. 

Assuming the stored wavelet coefficients vector for the subject *i* is represented as $X_{e}^{i}\left(w\right)=\left(D_{1}^{i},\;D_{2}^{i}\ldots \;D_{P}^{i}\right)$, where P is the decomposed level, a query subject j has a feature vector $X_{q}^{j}\left(w\right)=\left(D_{1}^{j},\;D_{2}^{j},\;\ldots \;D_{P}^{j}\right)$. The template matching classifier calculates the distance between the wavelet coefficients by:

(6) $$WDIST\left(i\right)=\overset{P}{\underset{p=1}{\sum }}\frac{\left||D_{p}^{i}-D_{p}^{j}|\right|}{\max\left(\left|D_{p}^{i}\right|\right)}$$

The smallest wavelet distance *WDIST*(*i*) indicates the identified subject *i*. 

In summary, the proposed two-stage classifier offers two advantages. First, it eliminates the concern from previous studies in which the subject verification accuracy of a conventional 1-to-N matching classifier tends to worsen when the subject size N gets too large [34]. The proposed algorithm eliminates this performance degradation concern as only 1-to-K template matching is needed, where K≪N. Second, the proposed algorithm significantly reduces the computation load. 

## 5. Results and Discussion

### 5.1. Single Random Forest Classifier 

**Figure 11:**
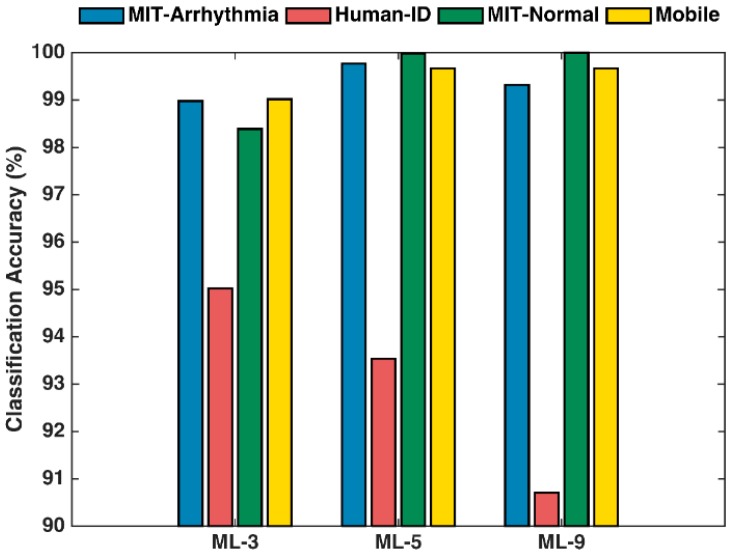
ECG biometric patient verification accuracy using random forest classifier only. ML-3 represents the features extracted from the Q, R, and S points; ML-5 represents the features extracted from the P, Q, R, S, and T points; and ML-9 further includes the wave boundaries P_on_, P_off_, T_on_, and T_off_ (according to Table 2).

The accuracy of machine learning classifier depends on the accurate detection of ECG fiducial points, as well as the features extracted from those fiducial points. For performance comparison purposes, we first assume that a single random forest algorithm is used and would be first independently evaluated in order to explore how the subject verification accuracy changes when the ECG features are extracted from different combinations of fiducial points. Following the above discussions, three cases listed in Table 2 are evaluated in this study to support our hypothesis that the local maximum/minimum fiducial points (P, Q, R, S, and T) are sufficient enough for the random forest classifier. Figure 11 shows the subject verification accuracy results for the three cases, where machine learning ML-3 represents the features extracted from the Q, R, and S points; ML-5 represents the features extracted from the P, Q, R, S, and T points; and ML-9 further includes the wave boundaries P_on_, P_off_, T_on_, and T_off_ (according to Table 2).

Four individual datasets are evaluated independently. It is observed that three datasets, MIT-arrhythmia, MIT-normal, and mobile ECG show a high accuracy (>99%) for both ML-5 and ML-9 scenarios. ML-9 shows slightly better accuracy than ML-5 for MIT-normal dataset. For the other datasets (MIT-arrhythmia and mobile ECG), the ML-9 does not show improvement over the ML-5. This is possibly due to the unreliable detections of the wave boundaries. The PhysioNet Human-ID dataset shows a totally different performance. ML-3 gives the best accuracy, while the ML-5 and ML-9 results degrade in performance. Further investigation of the PhysioNet Human-ID dataset indicates that there are lots of irregular ECG shapes, resulting in unreliable fiducial point detection. Overall, it is concluded that ML-5 gives the best performance among the four datasets, which confirms our hypothesis and is therefore used for the final system solution.

### 5.2. Single Wavelet Distance Classifier 

To obtain the best performance for the one-to-many template matching classifier based on wavelet coefficients while maintaining a low computational load, we investigate the subject verification accuracy of the template matching classifier using wavelet coefficients derived from three sets of decomposition levels, *S*1 = {D1, D2, D3, D4, D5}, *S*2 = {D2, D3, D4, D5}, and *S*3 = {D3, D4, D5} for all four individual ECG databases. Our hypothesis is that the optimized wavelet coefficients are obtained from those frequency sub-bands (see Table 3) within the ECG frequency range of 2–50 Hz, considering the fact that ECG signal is pre-processed by a bandpass filter from 2 to 50 Hz.

**Figure 12:**
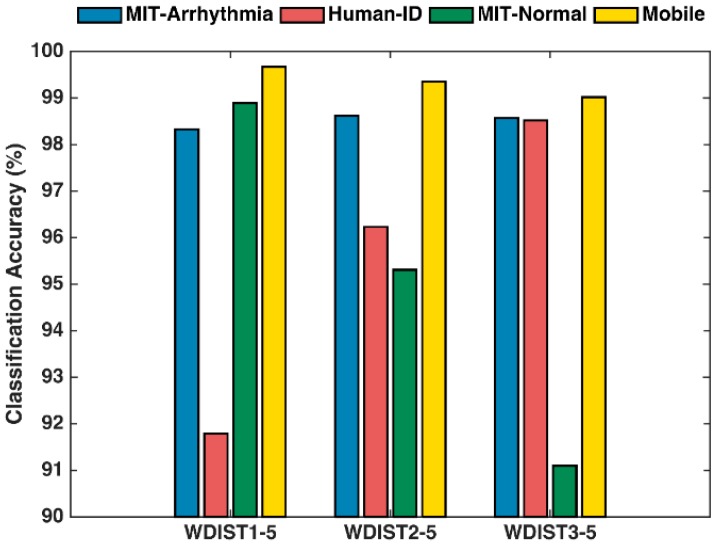
ECG biometric patient verification accuracy using 1-to-N classifier only. Three combinations of wavelet distance (WDIST) coefficients are presented. WDIST1-5: D1 to D5; WDIST2-5: D2 to D5; and WDIST3-5: D3 to D5.

Figure 12 presents the subject verification accuracy results using the 1-to-N template matching wavelet distance (WDIST) classifier with the three sets *S*1, *S*2, and *S*3 defined above. For the PhysioNet Human-ID dataset, the sampling rate is 500 Hz. The set *S*3 contains the ECG frequency range (2–50 Hz) and therefore gives the best performance. For the MIT-normal dataset, the set *S*1 contains the ECG frequency range due to the sampling rate of 128 Hz. Therefore, the performance degrades when less wavelet coefficients (set *S2, and set S3*) are included. For other two datasets with sampling rates 360 Hz and 400 Hz, on average, the set *S*2 gives the best performance.

### 5.3. Two-Stage Classifier 

**Figure 13:**
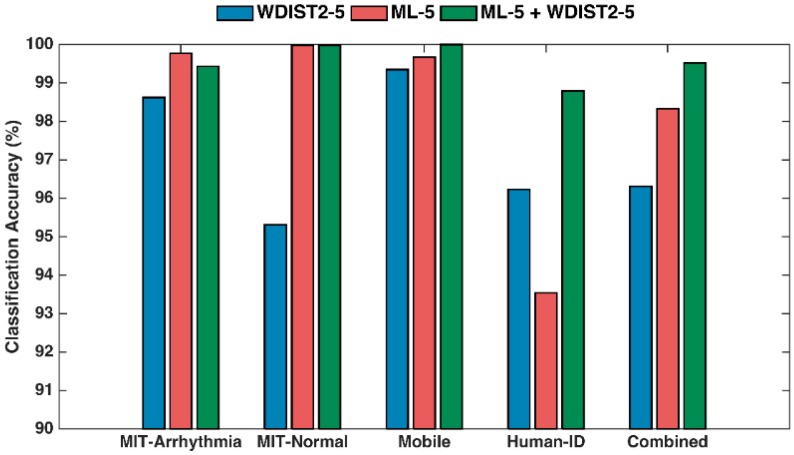
Comparisons of classifiers: the machine learning (ML-5), the one-to-many template matching (WDIST2-5), and the proposed two-stage classifiers (ML-5 + WDIST2-5) on the four individual datasets and the combined large dataset.

Based on the results from Figure 11 and Figure 12, further performance optimization is applied to utilize a probabilistic approach that combines the random forest method with the wavelet distance template matching classifier. The results are presented in Figure 13.

The proposed two-stage cascaded algorithm is evaluated on the four individual datasets and also on the combined dataset to demonstrate its ability to support the real-life practicality of ECG biometric verification. The probability threshold value is optimized as 0.15 for pre-determining the candidate subjects to be applied to the 1-to-K template matching classifier. The results in Figure 13 indicate that the two-stage cascaded classifier achieves an overall better accuracy than each individual classifier. For instance, for the combined dataset with 184 subjects, the WDIST2-5 and ML-5 achieves 96.31% and 98.33% subject verification accuracies respectively, while the cascaded two-stage classifier achieves a 99.52% subject verification accuracy. It should be noted that the unified dataset includes subject ECG measured from mobile phones, subject ECG measured in the presence of arrhythmia and subject ECG data measured 2–20 times over a 6-month period. Therefore, the high subject verification accuracy of 99.52% indicates the robustness of our proposed algorithm. In our study, the subject verification accuracy is defined as the number of subjects being correctly classified divided by the total number of subjects being tested. This leads to a small error rate of 0.48% where a subject is incorrectly classified. In our simulation, it is assumed that one subject is represented by only one ECG complex. In reality, the test subject could easily record more heartbeats (e.g., >10 heart beats) at a time to be used for subject verification. According to binomial theorem, using more than one heartbeat would further improve the subject verification rate over the results presented in this paper. 

From Figure 13, it can also be seen that the achieved accuracy improvement by the two-stage classifier depends on the quality of ECG data acquired. For instances, when ECG data is taken multiple times over a 6-month period, the proposed two-stage classifier achieves a 98.79% accuracy, better than the 93.54% from random forest alone and 96.23% from 1-to-N template matching alone. However, for subject ECG data that were taken from a single time test, such as MIT-BIH arrhythmia, MIT-BIH normal sinus rhythm, and ECG data from mobile phones, a high verification accuracy of better than 99% can be achieved using the single random forest classifier or the single 1-to-N wavelet distance measure classifier. Therefore, it is understandable that the improvement from the two-stage classifier over each single stage classifier would not be very obvious. 

Last, but not least, it is important to compare the performance of the proposed two-stage classifier to what was reported in literature. Due to the fact that many ECG studies use different datasets from different sources, and often, the data pre-processing technique, feature extraction and classification methods might all be different, a rigorous performance comparison might be impractical. However, the classification accuracy performance reported in literature can still be used as a benchmark and provide a relative reference for our study. Taking MIT-BIH ECG database as examples, Fatemian et al. [44] used the template matching approach for ECG subject identification, and reported an average of 99.61% verification accuracy when evaluated with MIT-BIH normal sinus rhythm database; Zokaee et al. [45] proposed using Mel-Frequency Cepstrum Coefficients (MFCC) for ECG feature extraction and k-Nearest Neighbors (KNN) for classification, and achieved 100% identification accuracy for MIT-BIH normal sinus rhythm database and 89% for ECG data gathered from 50 subjects in a hospital with three records of different times. Sasikala et al. [46] tested the feature extraction approach on ECG verification performance using the MIT-BIH arrhythmia database and achieved 99.0% accuracy; Zeng et al. [47] implemented the reduced binary pattern template matching on MIT-BIH arrhythmia and normal sinus rhythm databases and achieved a success rate of 95.79% and 90.19% separately. As a comparison, our proposed two-stage classification algorithm achieved a 99.43% accuracy for the MIT-BIH arrhythmia database and 99.98% for the MIT-BIH normal sinus rhythm database. These results further demonstrate the robustness and efficiency of the proposed two-stage classifier.

## 6. Conclusions

The main goal of this study is to facilitate the application of mobile ECG for biometric subject verification for applications where data security is demanding. Compared to the ECG data measured from a multi-lead clinical setup, the mobile ECG signals suffer from a poorer signal-to-noise ratio, baseline drift due to human respiration or motion artifacts from finger to electrode pad contact, as well as power line interferences. To overcome those challenges, a new two-stage ECG biometric verification system is proposed by combining probabilistic random forest method with wavelet distance measure. The motivation behind this approach is to decrease the environmental interference into a minimum effect on the verification accuracy. The proposed approach consists of two stages: (1) ECG fiducial features are first applied to a random forest ensemble learning classifier; (2) after the first stage classifier, only few candidates with probability higher than the threshold are applied to the template matching classifier. Compared with either the random forest classifier with ECG fiducial feature extraction, or the template matching classifier using wavelet distance measure only, the proposed hybrid approach is more robust against the environmental variations while still maintaining a low computational load.

The investigations of the robustness and effectiveness of the proposed two-stage algorithm are performed over four individual datasets. In addition, in order to simulate the real application environment where the sampling frequency of ECG signals might be different, a combined dataset is created using up/down sampling technique. Overall, for the combined 184 subjects, the proposed two-stage classifier achieves a total of 99.52% subject verification accuracy, better than the 98.33% accuracy from random forest alone and 96.31% accuracy from wavelet distance measure algorithm alone. It is also noted that the proposed two-stage algorithm is particularly effective when ECG data is acquired multiple times over a long time span. The evaluation results support that ECG signals can be potentially used for human biometric recognition using biosensors embedded into mobile devices. Furthermore, this algorithm can be adapted with dual ECG/fingerprint scanning to provide a highly reliable solution for a wide range of applications such as biosecurity and cybersecurity. 

## Figures and Tables

**Table 1:** Summary of the datasets used in this study. MIT-BIH: MIT-Beth Israel Hospital.

Data Sources	Subject Type	Subject Quantity	Number of Recordings	Sampling Rate
Mobile Phone	Healthy	30	Single	400 Hz
PhysioNet Human-ID	Age between 13 and 75	89	2–20 times over 6 months	500 Hz
MIT-BIH	Arrhythmia	47	Single	360 Hz
MIT-BIH	Normal sinus rhythm	18	Single	128 Hz
Mix of above	Mix of above	184	Mix of above	360 Hz

**Table 2:** Summary of extracted fiducial features. ML: machine learning; ML-N: using N fiducial points in ML.

Method	Fiducial Points	Temporal	Amplitude	Angle
ML-9	P, Q, R, S, T, P_on_, P_off_, T_on_, T_off_	T_1_ to T_15_	V_1_, V_2_, V_3_, V_4_, V_5_, V_6_, V_7_	A_1_ to A_6_
ML-5	P, Q, R, S, T	T_3_ to T_6_, T_11_, T_12_, T_15_	V_1_, V_2_, V_3_, V_4_, V_7_	A_3_ to A_5_
ML-3	Q, R, S	T_3_, T_4_	V_1_, V_2_, V_7_	A_5_

**Table 3:** Number of wavelet coefficients at each frequency sub-band.

Decomposition Level (*p* = 1 … 5)	Frequency Bandwidth *F_p_* (Hz)	Number of Wavelet Coefficients
D1	90 to 180	144
D2	45 to 90	72
D3	22.5 to 45	36
D4	11.25 to 22.5	18
D5	6.75 to 11.25	9
A5	0–6.75	9

## References

[1] Patel S., Park H., Bonato P., Chan L., Rodgers M. (2012). A review of wearable sensors and systems with application in rehabilitation. J. Neuroeng. Rehabil..

[2] Wang C., Wang Q., Ren K., Lou W. Privacy-Preserving Public Auditing for Data Storage Security in Cloud Computing. Proceedings of the INFOCOM.

[3] Boulos M.N.K., Wheeler S., Tavares C., Jones R. (2011). How smartphones are changing the face of mobile and participatory healthcare: An overview, with example from eCAALYX. Biomed. Eng. Online.

[4] Jain A., Bolle R., Pankanti S. (2006). Biometrics: Personal Identification in Networked Society.

[5] Israel S.A., Irvine J.M., Cheng A., Wiederhold M.D., Wiederhold B.K. (2005). ECG to identify individuals. Pattern Recogn..

[6] Shen T.W., Tompkins W.J., Hu Y.H. One-lead ECG for identity verification. Proceedings of the Second Joint Conference 24th Annual International Conference of the Engineering in Medicine and Biology Society, Annual Fall Meeting of the Biomedical Engineering Society.

[7] Biel L., Pettersson O., Philipson L., Wide P. (2001). ECG analysis: A new approach in human identification. IEEE Trans. Instrum. Meas..

[8] Irvine J.M., Wiederhold B.K., Gavshon L.W., Israel S.A., McGehee S.B., Meyer R., Wiederhold M.D. (2001). Heart rate variability: A new biometric for human identification. Proceedings of the International Conference on Artificial Intelligence (IC-AI’01).

[9] Kyoso M., Uchiyama A. Development of an ECG identification system. Proceedings of the 23rd Annual International Conference of Engineering in Medicine and Biology Society.

[10] Chan A.D., Hamdy M.M., Badre A., Badee V. (2008). Wavelet distance measure for person identification using electrocardiograms. IEEE Trans. Instrum. Meas..

[11] Shen T.W.D., Tompkins W.J., Hu Y.H. (2010). Implementation of a one-lead ECG human identification system on a normal population. J. Eng. Comput. Innov..

[12] Wübbeler G., Stavridis M., Kreiseler D., Bousseljot R.D., Elster C. (2007). Verification of humans using the electrocardiogram. Pattern Recogn. Lett..

[13] Agrafioti F., Hatzinakos D. Fusion of ECG sources for human identification. Proceedings of the 2008 ISCCSP 2008 3rd International Symposium on Communications, Control and Signal Processing.

[14] Singh Y.N., Gupta P. (2009). Biometrics method for human identification using electrocardiogram. Proceedings of the International Conference on Biometrics.

[15] Agrafioti F., Hatzinakos D. ECG based recognition using second order statistics. Proceedings of the CNSR 2008 6th Annual Communication Networks and Services Research Conference.

[16] Irvine J.M., Israel S.A. (2009). A sequential procedure for individual identity verification using ECG. EURASIP J. Adv. Signal Process..

[17] Ince T., Kiranyaz S., Gabbouj M. (2009). A generic and robust system for automated patient-specific classification of ECG signals. IEEE Trans. Biomed. Eng..

[18] Martis R.J., Acharya U.R., Min L.C. (2013). ECG beat classification using PCA, LDA, ICA and discrete wavelet transform. Biomed. Signal Process. Control.

[19] Lin C.H. (2008). Frequency-domain features for ECG beat discrimination using grey relational analysis-based classifier. Comput. Math. Appl..

[20] Daamouche A., Hamami L., Alajlan N., Melgani F. (2012). A wavelet optimization approach for ECG signal classification. Biomed. Signal Process. Control.

[21] Saini I., Singh D., Khosla A. (2013). QRS detection using K-Nearest Neighbor algorithm (KNN) and evaluation on standard ECG databases. J. Adv. Res..

[22] Christov I., Gómez-Herrero G., Krasteva V., Jekova I., Gotchev A., Egiazarian K. (2006). Comparative study of morphological and time-frequency ECG descriptors for heartbeat classification. Med. Eng. Phys..

[23] Wan Y., Yai J. A neural network to identify human subjects with electrocardiogram signals. Proceedings of the World Congress on Engineering and Computer Science.

[24] Li M., Narayanan S. Robust ECG biometric by fusing temporal and cepstral information. Proceedings of the 2010 20th International Conference on Pattern Recognition (ICPR).

[25] Goldberger A.L., Amaral L.A., Glass L., Hausdorff J.M., Ivanov P.C., Mark R.G., Mietus J.E., Moody G.B., Peng C.K., Stanley H.E. (2000). Physiobank, physiotoolkit, and physionet components of a new research resource for complex physiologic signals. Circulation.

[26] Moody G.B., Mark R.G. (2001). The impact of the MIT-BIH arrhythmia database. IEEE Eng. Med. Biol. Mag..

[27] Martínez J.P., Almeida R., Olmos S., Rocha A.P., Laguna P. (2004). A wavelet-based ECG delineator: Evaluation on standard databases. IEEE Trans. Biomed. Eng..

[28] Hu Y.H., Palreddy S., Tompkins W.J. (1997). A patient-adaptable ECG beat classifier using a mixture of experts approach. IEEE Trans. Biomed. Eng..

[29] Maglaveras N., Stamkopoulos T., Diamantaras K., Pappas C., Strintzis M. (1998). ECG pattern recognition and classification using non-linear transformations and neural networks: A review. Int. J. Med. Inform..

[30] Prasad G.K., Sahambi J.S. Classification of ECG arrhythmias using multi-resolution analysis and neural networks. Proceedings of the TENCON 2003 Conference on Convergent Technologies for the Asia-Pacific Region.

[31] De Chazal P., O’Dwyer M., Reilly R.B. (2004). Automatic classification of heartbeats using ECG morphology and heartbeat interval features. IEEE Trans. Biomed. Eng..

[32] Salahuddin L., Kim D. Detection of acute stress by heart rate variability (HRV) using a prototype mobile ECG sensor. Proceedings of the International Conference on Hybrid Information Technology.

[33] Wang Y., Plataniotis K., Hatzinakos D. Integrating Analytic and Appearance Attributes for Human Identification from ECG signals. Proceedings of the IEEE Biometrics Symposium 2006.

[34] Odinaka I., Lai P.H., Kaplan A.D., O’Sullivan J.A., Sirevaag E.J., Rohrbaugh J.W. (2012). ECG biometric recognition: A comparative analysis. IEEE Trans. Inf. Forensics Secur..

[35] Kailanto H., Hyvarinen E., Hyttinen J. Mobile ECG measurement and analysis system using mobile phone as the base station. Proceedings of the 2008 Second International Conference on Pervasive Computing Technologies for Healthcare.

[36] Ottenbacher J., Kirst M., Jatoba L., Huflejt M., Grossmann U., Stork W. Reliable motion artifact detection for ECG monitoring systems with dry electrodes. Proceedings of the 2008 30th Annual International Conference of the IEEE Engineering in Medicine and Biology Society.

[37] Szczepański A., Saeed K. (2014). A mobile device system for early warning of ECG anomalies. Sensors.

[38] Sufi F., Khalil I., Habib I. (2010). Polynomial distance measurement for ECG based biometric authentication. Secur. Commun. Netw..

[39] Zhang J., Hu X., Liu X., Dong J. A framework for ECG morphology features recognition. Proceedings of the 2010 IEEE 23rd International Symposium on Computer-Based Medical Systems (CBMS).

[40] Chernenko S. (2012). ECG Processing-R-Peaks Detection, Librow TM. www.librow.com.

[41] Pan J., Tompkins W.J. (1985). A real-time QRS detection algorithm. IEEE Trans. Biomed. Eng..

[42] Vandenberk B., Vandael E., Robyns T., Vandenberghe J., Garweg C., Foulon V., Willems R. (2016). Which QT Correction Formulae to Use for QT Monitoring?. J. Am. Heart Assoc..

[43] Nyquist H. (2002). Certain topics in telegraph transmission theory. Proc. IEEE.

[44] Fatemian S.Z., Hatzinakos D. A new ECG feature extractor for biometric recognition. Proceedings of the 16th International Conference on Digital Signal Processing.

[45] Zokaee S., Faez K. (2012). Human Identification Based on Electrocardiogram and Palmprint. Int. J. Electr. Comput. Eng..

[46] Sasikala P., Wahidabanu R.S.D. (2010). Identification of individuals using electrocardiogram. Int. J. Comput. Sci. Netw. Secur..

[47] Zeng F., Tseng K.K., Huang H.N., Tu S.Y., Pan J.S. A new statistical-based algorithm for ECG identification. Proceedings of the Eighth International Conference on Intelligent Information Hiding and Multimedia Signal Processing (IIH-MSP).

